# The Difference in Gold Foils

**Published:** 1860-07

**Authors:** 


### ARTICLE V.

### *The Difference in Gold Foils.*

So much has been said and written about gold foil, that perhaps it would be quite impossible to say anything new, we therefore merely offer our opinion upon the use of such foils in our hands as it has been our fortune to obtain.

Of Abbey's foil, as an even and reliable article, as far as we have tested, too much cannot be said. The lower the number of this manufacturer the better has been the working quality, being more yielding in its introduction into cavities, condensing to a more absolute solid, and, I think, welding with less pressure. Of course adapting itself better to the walls of the cavity, the most desirable quality yielding a finish equal to the best of higher numbers and deemed requiring less time and I may say less manipulative skill. Its great and most peculiar merit is its even, unchanging character, each lot being a counterpart of the previous one, and upon this has the good character of this gold been founded, and not that it is capable of doing more than the make of others will do, but because it is always alike.

With Morgan's foil his higher numbers work better than the lower. I have found Morgan's No. 10 adhesive to work quite well, indeed of all foils I ever used, a lot of his No. 10 was certainly the best, for a similar lot of which I would gladly pay two prices. But it is to be much regretted that the equality of character of this foil is not so prominent as that of the former, or else there would soon be none other used. But it has varied from the very best to the quite indifferent. In some books there will be found that soft evenness to the pressure of an instrument, adhering to surfaces of cavity without becoming inflexible, enabling one to feel an entire command over every operation he may undertake, and which can never be experienced in foil that is resisting and harsh, springing and breaking under the slightest force.

The connection between an operator and the foil he is using, can only be understood by one who for days and years has been earnestly and heartily trying to do whatever he wills with it, which enables him to detect, as it were, a vital connection between the instrument, foil, tooth and himself, so intimate as to indicate the differences and changes as belong to the very highest order of instinct acting upon matter.

Of Jones & White's foil we have not had the good fortune to use sufficient of it to pass an opinion upon it, but what we have used of it, would place it among the medium qualities, and as we write only our own experience we do not repeat many good things we have heard.

Watts & Co's adhesive sponge gold foil will occasionally produce results almost incomparable, but a fresh lot will prove useless or nearly so, as it can only be used in unseen corners, where hard things are alone bearable, and is often slippery and very obstinate, impossible to weld, and retaining its position only by opposing sides and constant pressure. We say this with much regret, for we have used of it, foil better than which never was made, and have made fillings of it as beautiful as our ability could effect, thinking that for the future our troubles were at an end, but, lamentable fact, the next lot would let us down to the other extreme.

Our own preference is for foil made in this way. It perhaps is quite as expeditious, can be absolutely pure, and its structure, when made, must be better adapted for the operation of filling than by a purely lamellated one.

When good it is used with such pleasant ease, becoming solid only when the cavity is full, and requiring the most delicate pressure, applied throughout the filling, or when a nerve is exposed it casehardens so readily over it, presenting when finished a perfectly solid and burnished surface. We believe its adhesive qualities are different and distinct from the common foils annealed, enabling it to be used in all cases alike admirably. For instance, in building a crown, sponge gold itself is not better, and for filling deep cavities it can be made as solid as a bullet, adhering closely to the walls, so much so as to appear to work itself into the very structure of the dentine, whilst annealed foil works with such increasing sluggishness or obstinacy as renders it the most uncertain and difficult at times in the hands of the best operators. Indeed if the cavity is not always shaped so as to retain the gold from the most decided enlargements within, it will frequently come out in a solid mass, without the least attachment to the tooth from adhesion, or from pressure, owing to its great tendency to weld with its own particles only.

Let Watts & Co. make an invariably good article and they would have no difficulty of supplying the best operators entirely. Abbey's foil, although not so capable as the best sponge foil, is always better in use, because it is even in its peculiar qualities, and the others vary, the operator not knowing how to rely upon them ; I would like also to say Morgan's, but the former is more constant than the latter, although the latter is sometimes better than the former. We have used foil made by many other manufacturers but too little to judge of their respective merits. Merely good foil is made everywhere, but the best in our opinion is found sometimes in the manufactures of Abbey, Morgan and Watts, with the restrictions above made according to the experience and practice of

TWELVE YEARS.

